# Psychometric properties of the Short-Form McGill Pain Questionnaire (SF-MPQ) in adult Mexican cancer patients with chronic pain

**DOI:** 10.1017/S1478951524001731

**Published:** 2025-01-13

**Authors:** Luis Alberto Mendoza-Contreras, Benjamín Domínguez Trejo, María del Rocío Guillén Núñez, David Alberto Rodríguez Medina, Xolyanetzin Montero Pardo, Tania Estapé, Oscar Galindo Vázquez

**Affiliations:** 1 Facultad de Psicología, Universidad Nacional Autónoma de México, Mexico City, Mexico; 2 Pain Clinic, Instituto Nacional de Cancerología, INCan, Mexico City, Mexico; 3 Unidad Iztapalapa, Department of Sociology, Social Sciences and Humanities Division, Universidad Autónoma Metropolitana, Mexico City, Mexico; 4 Facultad de Psicología, Universidad Autónoma de Sinaloa, Culiacan, Mexico; 5 Coordinadora de Psicooncología, FEFOC Fundación Barcelona, Barcelona, Spain; 6 Department of Psycho-Oncology Service, Instituto Nacional de Cancerología, INCan, Mexico City, Mexico

**Keywords:** Cancer, Chronic pain, Psychometric properties, SF-MPQ, Mexican population

## Abstract

**Background:**

Pain is a frequent symptom in cancer patients (CP), and its multidimensional assessment is essential for a comprehensive approach and to establish clinical prognoses. The Short-Form McGill Pain Questionnaire (SF-MPQ) is an internationally recognized tool for the multidimensional assessment of pain, both in clinical and research settings. However, no studies have been reported in Latin America that determine its psychometric properties in CP and chronic pain.

**Objectives:**

To determine the psychometric properties of the SF-MPQ in adult Mexican cancer patients with chronic pain.

**Methods:**

An instrumental design was used with a non-probabilistic convenience sample of 222 cancer patients treated at the pain clinic of a tertiary care hospital. Analyses were conducted to evaluate factorial structure (exploratory and confirmatory factor analysis [CFA]), reliability (internal consistency), measurement invariance, and criterion validity (concurrent and divergent).

**Results:**

CFA verified a 9-item structure divided into 2 factors: (1) Affective-Nociceptive and (2) Neuropathic. A global Cronbach’s alpha coefficient of .82 and a global McDonald’s Omega index of .82 were identified. Configural, metric, and scalar invariance (ΔCFI ≤ .01; ΔRMSEA ≤ .015) were confirmed regarding the sex variable. Finally, the SF-MPQ showed a positive correlation with the Numerical Rating Scale (rho = .436, *p*< .01) and a negative correlation with the EORTC-QLQ C30 (rho = −.396, *p*< .01).

**Significance of results:**

The Mexican version of the SF-MPQ presented adequate psychometric properties and fit indices, making it a valid and reliable instrument for use in clinical and research settings in Mexico. Its use is recommended for the comprehensive assessment of pain in oncology in Mexico, as it allows for the understanding of pain characteristics beyond intensity, guiding the establishment of clinical prognoses.

## Introduction

Cancer is a significant public health issue globally (Sánchez et al. 2022). In 2022, approximately 19.9 million new cases were reported worldwide, and in Mexico, around 207,154 new cases and 96,210 deaths (International Agency for Research on Cancer. GLOBOCAN 2022a, 2022b). In this context, pain is one of the most frequent symptoms in cancer patients (CP) (Davis et al. 2021) and a variable that directly impacts their quality of life (Decoster et al. 2019).

Pain is defined as an unpleasant sensory and emotional experience associated with, or similar to that associated with, actual or potential tissue damage (International Association for the Study of Pain in Raja et al. 2020). It comprises 3 main dimensions: (1) *Sensory-discriminative*, which encompasses the quality, location, duration, and intensity of pain; (2) *Motivational-affective*, which includes subjective aspects such as suffering, aversion, dislike and experienced emotional changes; and (3) *Cognitive-evaluative*, which comprises the person’s previous experiences and response strategies (Chóliz 1994; Pinzón et al. 2019). In this regard, the close bidirectional relationship between the sensory dimension and the emotional response suggests that pain intensity significantly impacts the emotional state of CP, and vice versa (Cramer et al. 2018; Kang and Choi 2019; Schreier et al. 2019).

To effectively assess pain in CP, it is essential to use instruments that are valid and reliable (Gauthier et al. 2014). In this sense, the most commonly measured dimension is intensity, and even though the Mexican Consensus on Cancer Pain (Allende et al. 2016) includes unidimensional scales such as the Visual Analog Scale (VAS) (Guevara-López et al. 2005) and the Numerical Analog Scale (Flaherty 1996), it emphasizes the need to adopt a multidimensional approach for the initial assessment and follow-up of pain in CP, which can be relevant for establishing clinical prognoses (Mendoza-Contreras et al. 2024).

To assess the sensory-discriminative, motivational-affective dimensions, and pain intensity, Ronald Melzack developed the Short-Form McGill Pain Questionnaire (SF-MPQ) (Melzack 1987). Over the past 4 decades (Main 2016), this instrument has been widely used as an internationally recognized assessment tool in clinical and research settings (Bourzgui et al. 2021; Oliveira et al. 2021). Additionally, it has been applied in recent studies with breast cancer patients undergoing surgery (Shiraishi et al. 2022; Xia et al. 2022), head and neck cancer (Lou et al. 2021), and patients treated in pain and palliative care clinics (Anagnostopoulos et al. 2023).

In Mexico, there is a need for a valid, reliable, and psychometrically adequate instrument for the multidimensional assessment of pain in CP. As far as we know, there is no report of the psychometric properties analysis of the SF-MPQ in CP in Latin America. Therefore, the objective of this study was to determine the psychometric properties of the SF-MPQ in adult Mexican cancer patients with chronic pain.

## Method

### Participants

This study utilized a convenience sample obtained from the Pain Clinic at the National Cancer Institute of Mexico (INCan) between April 13 and September 5, 2023. An instrumental design was employed (Montero and León 2005). The sample size was determined based on current recommendations for evaluating the psychometric properties of an instrument, with a minimum of *n* = 200 participants (Lloret-Segura et al. 2014). The eligibility criteria for participation in the study were as follows:

- *Inclusion Criteria*: Confirmed oncological diagnosis, either first-time or subsequent visit to the pain clinic, undergoing active oncological treatment, aged 18 years or older, any clinical stage and Karnofsky Performance Status ≥40.
- *Exclusion Criteria*: Experiencing severe pain, fatigue, nausea, or any other symptom expressed as severe that would prevent the participant from completing the scales and cognitive impairment preventing scale completion.
- *Elimination Criteria*: Participant decides to discontinue participation during the completion of the instruments.

### Instruments

- *Identification Form.* A participant identification form was designed to collect sociodemographic and clinical data, such as age, sex, education level, place of residence, and information related to pain characteristics (e.g., number of anatomical areas with different pains reported at the time of assessment, anatomical area of main pain, duration of main pain), cancer diagnosis, clinical stage, medical treatment, and functionality level.
- *Numerical Rating Scale (NRS)*. Participants were asked to rate the intensity of their pain at the time of assessment from 0 to 10 (where 0 is no pain and 10 is the worst pain imaginable) (Safikhani et al. 2018).
- *Short-Form McGill Pain Questionnaire (SF-MPQ).* Developed by Melzack (1987), this self-report instrument comprises 15 items that assess the sensory (11 items) and affective dimensions of the pain experience (4 items). Additionally, it includes a VAS and an indicator of present pain intensity; the 15 descriptors of the 2 dimensions are rated on a Likert scale (0 = no pain, 1 = mild, 2 = moderate, and 3 = severe), while the VAS score (item 16) ranges from 0 (no pain) to 100 (worst possible pain) and the score for item 17 ranges from 0 (no pain) to 5 (unbearable).
- *European Organization for Research and Treatment of Cancer Quality of Life Questionnaire (EORTC QLQ-C30).* Developed by Aaronson et al. (1993) and validated in Mexico by Oñate-Ocaña et al. (2009), this questionnaire consists of 30 items scored on a 1–4 ordinal scale and 2 items scored from 1 to 7. It is divided into 3 dimensions: (1) Functional, including physical, role, cognitive, emotional, and social functioning; (2) Symptoms, covering fatigue, pain, nausea, and vomiting; and (3) Global health and quality of life. The questionnaire has an internal consistency of α = 0.90 and concurrent validity with functional status according to the Karnofsky scale (*p*< .001).

## Procedure

### Pilot test

A pilot test was conducted with 20 adult cancer patients with chronic pain from the Pain Clinic at INCan to identify potential issues with the wording of items, instructions, and response options of the Spanish version of the SF-MPQ for Mexico (Koller et al. 2007). During this phase, no modifications were made to the instrument, but it was suggested that a healthcare professional administer the instrument to address any questions and provide examples of pain descriptors. Therefore, subsequent administration was conducted through interviews.

### Statistical analysis

Statistical analyses were performed using SPSS version 26, including means and standard deviations (SD), skewness, kurtosis, item-total correlations, and alpha if item deleted. Reliability was assessed through internal consistency (Cronbach’s alpha and McDonald’s omega). Sample adequacy indices of Kaiser–Meyer–Olkin (KMO) and Bartlett’s test of sphericity were verified for subsequent analyses. Factor structure and factor loadings were examined, as well as the percentage of explained variance using an exploratory factor analysis (EFA) with the principal axis factoring extraction method and Equamax rotation.

The SF-MPQ was analyzed using confirmatory factor analysis (CFA) models with AMOS version 24. Model quality was assessed using indices such as *χ*^2^ and *χ*^2^/*df* ratio, goodness-of-fit indices (GFI, NFI), comparative fit index (CFI), and root mean square error of approximation (RMSEA) (Byrne 2010; Ullman 2006). Multigroup factorial analysis was employed to analyze measurement invariance with respect to gender (male and female). Finally, Spearman correlations were used to obtain evidence of criterion validity (concurrent and divergent), with a significance level of *p*< .05.

## Results

### Sociodemographic and clinical characteristics

The sociodemographic and clinical characteristics of the 222 participants are shown in Table 1, with 69.4% being women and an average age of 53.16 years (SD = 12.40; range = 20–81 years).

**Table 1.** Sociodemographic and clinical characteristics of a sample of 222 participants with cancer and chronic pain

Age in years: X̅ = 53.16, *SD*= 12.40, range = 20−81					
Variable	*f*	%	Variable	*f*	%
**Sex**			**Residence**		
Woman	154	69.4	Downtown area	85	38.3
Man	68	30.6	Conurbation zone	69	31.1
**Educational status**			Rural zone	68	30.6
None	6	2.7	**Cancers by body location/system**		
Elementary school	61	27.5	Breast	44	19.8
Junior high school	62	27.9	Genitourinary	38	17.1
Senior high school	47	21.2	Hematologic/Blood	38	17.1
University	42	18.9	Gynecologic	28	12.6
Postgraduate and above	4	1.8	Digestive/Gastrointestinal	28	12.6
**Marital status**			Respiratory/Thoracic	11	5.0
Single	60	27.0	Head and neck	9	4.1
Married	93	41.9	Skin	13	5.9
Widowed	22	9.9	Musculoskeletal	7	3.2
Divorced/separated	21	9.5	Other	6	2.7
Free union	25	11.3	**Stage (TNM)^b^**		
Another	1	.5	I	9	5.8
**Occupation**			II	31	19.9
Homemaker	111	50.0	III	38	24.4
Employee	13	5.9	IV	78	50.0
Unemployed	58	26.1	**Metastasis**		
Self-employed	22	9.9	Yes	94	42.3
Professional	10	4.5	No	128	57.7
Retired	3	1.4	**Comorbidity**		
Another	5	2.3	Yes	103	46.4
**Mental health care throughout the lifespan**			No	119	53.6
Yes	93	41.9	**Number of comorbidities**		
No	129	58.1	One	51	49.5
**Type of mental health care**			Two	34	33.0
Psychology	62	66.7	Three	11	10.7
Psychiatry	6	6.5	Four	3	2.9
Both	25	26.9	Five	4	3.9

^a^=103 participants.

b =156 participants.

### Pain characteristics

The number of anatomical areas with different pains at the time of assessment was Mdn = 2, the intensity of the main pain (assessed with the NRS) was Mdn = 4, and the duration of the main pain was Mdn = 8 months (see Table 2).

**Table 2.** Pain characteristics of a sample of 222 participants with cancer and chronic pain

Numerical Rating Scale Mdn = 4, Anatomical areas with different pain reported at the time of assessment Mdn = 2, Time with primary pain Mdn = 8 months					
Variable	*f*	%	Variable	*f*	%
**Anatomical area of main pain**			**Cancer-related pain diagnosis**		
Head and neck	15	6.8	Yes	169	76.1
Thorax	19	8.6	No	26	11.7
Back	40	18.0	Not specified	27	12.2
Pelvis	21	9.5	**Type of cancer-related pain^a^**		
Upper limb	27	12.2	Bone cancer pain	25	14.8
Lower limb	62	27.9	Visceral cancer pain	23	13.6
Abdomen	23	10.4	Post-cancer surgery pain	17	10.1
Buttocks	15	6.8	Post-cancer medicine pain	17	10.1
**Temporality**			Mixed pain	62	36.7
Continuous	106	47.7	Another cancer-related pain	25	14.8
Flashing	116	52.3			

Mdn = Median.

a =169 participants.

### Descriptive evaluation of the items

The results obtained from the items, including frequency distribution, skewness, kurtosis, inter-item correlation indices, corrected homogeneity index (cHI), and contrasted extreme groups, are presented in Table 3.

**Table 3.** Descriptive evaluation of the SF-MPQ items

Item	Mean	Standard deviation	Range	High frequency options^a^	Floor or ceiling effects^a^	Asymmetry/Kurtosis^a^	Inter-item corr.	Item-scale total corr.	Extreme groups
1	0.84	1.029	0–3	53.1	53.1/9.0	0.83/−.67	.08/.27	0.32	.001
2	0.78	1.02	0–3	55.5	55.5/10.0	1.03/24	.01/.37	0.34	.001
3	0.61	1.083	0–3	72.5	72.5/12.8	1.43/.39	.19/.35	0.50	.001
4	1.03	1.071	0–3	43.6	43.6/11.8	0.52/1.09	.10/.34	0.42	.001
5	0.86	1.112	0–3	57.3	57.3/12.3	0.83/−.86	.04/.37	0.29	.001
6	0.42	0.843	0–3	77.3	77.3/4.3	1.87/−2.24	.07/.32	0.34	.001
7	1.02	1.14	0–3	48.8	48.8/14.7	0.55/−1.22	.01/.36	0.51	.001
8	1.46	1.109	0–3	32.7	28.0/20.9	−0.06/−1.35	.36/.56	0.55	.001
9	1.11	1.105	0–3	43.1	43.1/12.8	0.35/−1.33	.08/.40	0.46	.001
10	0.7	1.074	0–3	66.8	66.8/10.0	1.10/−.38	.07/.30	0.42	.001
11	0.64	1.079	0–3	70.6	70.6/11.4	1.29/.04	.04/.46	0.49	.001
12	1.33	1.176	0–3	37.0	37.0/20.9	0.12/–1.50	.08/.48	0.56	.001
13	0.25	0.682	0–3	86.3	86.3/2.4	2.72/6.47	.01/.25	0.31	.001
14	0.57	1.009	0–3	71.6	71.6/9.5	1.49/.73	.04/.43	0.44	.001
15	0.85	1.143	0–3	59.2	59.2/14.2	0.87/−.85	.08.46	0.56	.001

corr. = correlation.

a =percentage.

### Exploratory factor analysis

Items M1 (Throbbing), M5 (Cramping), M6 (Gnawing), M10 (Tender), and M13 (Sickening) were eliminated based on combined criteria, including frequency distribution (>50% in 1 response option), skewness and kurtosis (>1), item-item correlation (<.20), contrasted extreme groups (*p* > .05), communalities (<.50), and factor loadings (<.40). Table 4 shows the factor loadings obtained through EFA (with KMO = .843 and Bartlett’s test of sphericity: *χ*^2^(45) = 590.229, *p* < .001), revealing a 2-factor structure (Factor 1: Affective-Nociceptive and Factor 2: Neuropathic) that explained 39.89% of the total variance.

**Table 4.** Exploratory factor analysis (EFA) of the SF-MPQ

Cronbach´s alpha coefficient *α* = .82 *ω* **=**.82 Total explained variance = 39.82%	Factorial loading	
	Factor 1	Factor 2
M15. Punishing-cruel	0.713	
M12. Tiring-exhausting	0.677	
M8. Aching	0.634	
M11. Splitting	0.618	
M14. Fearful	0.537	
M9. Heavy	0.497	
M2. Shooting		0.722
M4. Sharp		0.5
M3. Stabbing		0.43
M7. Hot-burning		0.43
Cronbach´s alpha	*α* = .80	*α* = .65
Omega	*ω* **=**.81	*ω* **=**.65
Explained variance	26.19%	13.69%

### Internal consistency

The overall internal consistency obtained was *α* = .82 and *ω* = .82. For the Affective-Nociceptive factor, the values were *α* = .80 and *ω* = .65, and for the Neuropathic factor, they were *α* = .81 and *ω* = .65.

### Confirmatory factor analysis

**Figure 1.**
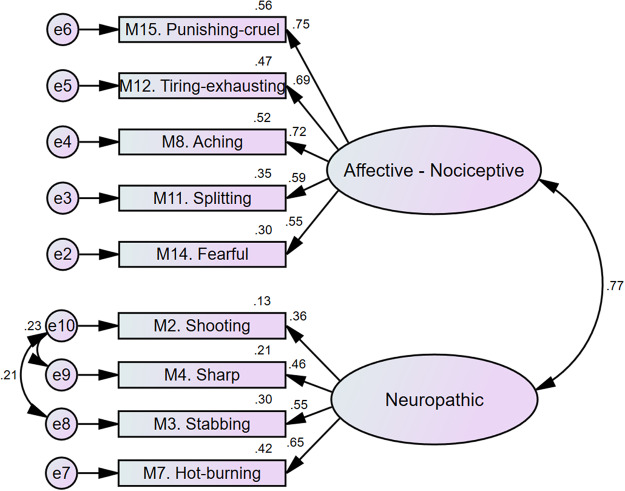
Two-factor first-order confirmatory factor analysis model with 9 items of the *SF-MPQ.*

A CFA was conducted to evaluate the fit of the final scale structure. Modification indices indicated the need to establish covariances between residuals, which were explored through different covariance models detailed in Table 5. Model 2, with 9 items, demonstrated the most adequate fit. The standardized factor coefficients along with the fit indices were satisfactory: *χ*^2^(24) = 43.532; CMIN/DF = 1.814; CFI = 0.960; NFI = 0.916; GFI = 0.962; AGFI = 0.928; SRMR = 0.043; RMSEA = 0.061 (0.030–0.089) (*p*< 0.001). This model is presented in Fig. 1, showing the standardized factor coefficients with the obtained fit indices.

**Table 5.** Fit indices obtained for each one of the tested models

Model	MI suggested adjustments made	*χ^2^ (gl)*	CMIN/DF	NFI	CFI	GFI	AGFI	SRMR	RMSEA
1	Model proposed by the EFA	77.183/ 34	2.270 (*p*< .001)	.872	.922	.939	.902	.057	.076 (.053/.098)
2	Deletion of item M9; association between e8 and e10, e9, and e10	43.532/24	1.814 (*p*< .001)	.916	.960	.962	.928	.043	.061 (.030 /.089)
Cut-off criteria			<3	>.90	>.95	>.90	>.90	<.05	<.08

MI: modification index; M9: Heavy; e8: residual error 8; e9: residual error 9; e10: residual error 10; *X*^2^ (gl): Chi-square (degrees of freedom); CMIN/DF: Chi-square ratio over degrees of freedom; NFI: normed fit index; CFI: comparative fit index; GFI: goodness-of-fit index; AGFI: adjusted goodness-of-fit index; SRMR: standardized root mean square residual; RMSEA: root mean square error of approximation per degrees of freedom.

### Measurement invariance

A multigroup CFA was conducted to test the measurement invariance of the SF-MPQ between groups defined by gender (women and men). The model tests included the configural invariance model (M1), the metric invariance model (M2), and the scalar invariance model (M3), all of which showed a good fit. However, the strict invariance model (M4) was significant, as shown in Table 6.

**Table 6.** Results of tests of measurement invariance by sex

Model	*X*^2^(gl)	CMIN/DF	CFI	RMSEA (CI 90%)	Model comparison	Δ ^2^	ΔCFI	ΔRMSEA
M1. Configural invariance	85.929 (48)	1.790	.926	.060 (.039–.080)				
M2. Metric invariance	98.203 (55)	1.786	.916	.060 (.040–.079)	M2 vs M1	12.273 (7) *p*= .092	−0.01	0
M3. Scalar invariance	113.699 (64)	1.777	.903	.059 (.041–.077)	M3 vs M2	15.496 (9) *p*= .077	−0.013	−0.001
M4. Strict invariance	140.658 (78)	1.803	.878	.060 (.044–.076)	M4 vs M3	26.959 (14) *p*= .046	−.025	0.001

### Criterion validity

Evidence of validity based on the relationship with other variables was obtained. As presented in Table 7, for concurrent validity with the NRS, positive and significant correlations were found between the Affective-Nociceptive Factor, the Neuropathic Factor, and the global scale of the SF-MPQ. In terms of divergent validity, negative and significant relationships were found between global quality of life and the Affective-Nociceptive Factor, the Neuropathic Factor, and the global scale of the SF-MPQ.

**Table 7.** Correlations between the SF-MPQ, NRS and EORTC-QLQ-C30 instruments

	TOTAL SF-MPQ	AN	N	VAS	PPI
NRS	.436**	.417**	.311**	.650**	.768**
*QLQ-C30 QoL*	−.396**	−.426**	−.238**	−.381**	−.350**

AN = Affective-Nociceptive; N = Neuropathic; VAS = Visual Analogue Scale; PPI = Present Pain Intensity; QoL = Quality of Life.

** *p*< .01.

## Discussion

The objective of this study was to determine the psychometric properties of the SF-MPQ in Mexican adult cancer patients with chronic pain. The SF-MPQ has been used in over 250 published studies; however, few have examined the core constructs it measures (Mason et al. 2008). In studies with cancer patients, it is common to include the original version in reports (Lou et al. 2021; Wiener et al. 2024; Xia et al. 2022), even though it is recommended to reassess the reliability and validity of instruments in the populations studied (Han et al. 2002). This study conducted in a Mexican population determined a bifactorial structure (Factor 1: Affective-Nociceptive and Factor 2: Neuropathic) through an EFA and CFA, different from the original model’s item grouping. A similar structure was previously reported in Asian-American cancer patients (Shin et al. 2007). Additionally, Mason et al. (2008) evaluated different models of the SF-MPQ, finding that the sensory and affective-sensory factors fit the data better than Melzack’s model.

This item grouping can be theoretically interpreted as follows. First, cancer-related chronic pain is mostly considered a mixed type of pain, both neuropathic and nociceptive (Bennett et al. 2019). Furthermore, the complexity of its characteristics, such as the number of anatomical areas with different pains reported at the time of assessment (Mdn = 2; range = 1–4), the baseline pain (47.7%) or intermittent pain (52.3%), the main pain’s anatomical area (lower extremities in first place [27.9%], followed by back [18.0%]), and oncological pain diagnoses (mixed pain in first place [36.7%], followed by bone pain [14.8%]) might influence the factorial structure (Hernán 2013).

Second, it is likely that cancer patients describing their pain with nociceptive characteristics (as if the painful area is about to burst or hurts) perceive it as more threatening (tiring-exhausting, fearful, and punishing-cruel) compared to those indicating neuropathic pain characteristics like electric shock or burning (Yoon and Oh 2018), who do not recognize these characteristics as pain but as another bodily sensation. Lastly, the cultural role influences the meanings of pain descriptors (Im and Chee 2001); for example, in our context, participants commonly associate the word “sensitive” with an emotional issue rather than hyperalgesia, besides experiencing emotions like frustration or anger instead of fear.

The elimination of items (M1. Throbbing, M5. Cramping, M6. Gnawing, M9. Heavy, M10. Tender, and M13. Sickening) was based on descriptive analyses of each item and the fit of the EFA and CFA (Bandalos et al. 2010; Ferrando and Anguiano-Carrasco 2010; Hair et al. 2006; Lloret-Segura et al. 2014). Although this 9-item version follows current recommendations on the use of brief and simple scales suitable for clinical settings (Ferrer-Peña et al. 2016), it is suggested that future studies replicate this factorial structure in cancer patients.

Adequate internal consistency indices were identified, with an overall Cronbach’s alpha of *α* = .82 and McDonald’s omega of *ω* = .82, which were below the range reported in previous studies (*α* = .85-α = .93; *ω* = .89-*ω* = .96) (Choi et al. 2015; Sandhu 2017; Terkawi et al. 2017). Nevertheless, both indices indicate good internal consistency (>.80) (George and Mallery 2003; Moral 2019).

On the other hand, the measurement invariance of a scale is a psychometric property that determines whether it measures the same latent construct in different subgroups of a sample, being essential for making valid group comparisons (Astudillo-García et al. 2022). In our study, the findings regarding measurement invariance were adequate. Overall, the results supported the good fit of the items to the 2 proposed factors for the SF-MPQ and showed that the factorial structure remains invariant regarding gender. The fit indices were adequate, except for 1 parameter in the strict invariance model; in this case, an unbiased invariance would be assumed (Dimitrov 2010), as strict invariance tests are considered too restrictive (Bentler 2004). Consequently, the scores could be predominantly comparable between the groups.

Regarding criterion validity, it was evaluated through correlations (positive, low to moderate) between the global SF-MPQ, its factors and indicators, and the NRS. Additionally, negative and statistically significant correlations were identified with the global quality of life scores of the EORTC QLQ-C30. These findings are consistent with the literature, which indicates that pain is related to quality of life and interferes with various aspects of the daily lives of cancer patients (Mendoza-Contreras et al. 2024).

Among the limitations, the absence of probabilistic sampling stands out. Additionally, factors such as the time elapsed since the prescribed pain medications were taken, the rescues, and/or interventional procedures could influence the underestimation of pain intensity and characteristics by patients at the time of assessment.

In conclusion, the Mexican version of the SF-MPQ presents adequate psychometric properties and fit indices, making it a brief, valid, and reliable multidimensional instrument for use in clinical and oncological pain research settings in Mexico. Furthermore, its use allows for the comparison of results at the national and international levels. It is suggested that future research on the SF-MPQ examine the proposed factorial structure, its relationship with other constructs such as pain catastrophizing, social support, and emotional symptoms, as well as the instrument’s ability to detect changes over time and with treatment.
